# Neurotropic Highly Pathogenic Avian Influenza A(H5N1) Virus in Red Foxes, Northern Germany

**DOI:** 10.3201/eid2912.230938

**Published:** 2023-12

**Authors:** Christine Baechlein, Sven Kleinschmidt, Dorothee Hartmann, Patricia Kammeyer, Anne Wöhlke, Tobias Warmann, Louise Herms, Bianca Kühl, Andreas Beineke, Peter Wohlsein, Timm Harder, Martin Runge

**Affiliations:** Lower Saxony State Office for Consumer Protection and Food Safety, Braunschweig/Hannover, Germany (C. Baechlein, S. Kleinschmidt, D. Hartmann, P. Kammeyer, A. Wöhlke, T. Warmann, L. Herms, M. Runge);; University of Veterinary Medicine Hannover Foundation, Hannover, Germany (B. Kühl, A. Beineke, P. Wohlsein);; Friedrich-Loeffler-Institut, Greifswald-Insel Riems, Germany (T. Harder)

## Abstract

In a 1-year survey of wild terrestrial predators in northern Germany, we found that 5 of 110 foxes were infected with contemporary avian influenza A(H5N1) viruses, forming a temporal cluster during January‒March 2023. Encephalitis and strong cerebral virus replication but only sporadic mammalian-adaptive viral polymerase basic 2 protein E627K mutations were seen.

Since emergence of the highly pathogenic avian influenza virus (HPAIV) H5 A/Goose/Guangdong/1/1996 (gs/GD) lineage in 1996, successors continue to circulate in waves around the world, leading to massive losses in wild bird and domestic poultry populations (*1*). Until 2020‒2021, gs/GD HPAIV infections in poultry holdings characteristically paralleled waterfowl migration patterns. Since then, this seasonality has virtually disappeared and gs/GD HPAIV, currently of subtype H5N1 assigned to clade 2.3.4.4b, are detected year-round in wild birds and poultry in Europe (*2**,**3*). The virus has been found at increasing frequency in domestic and wild living mammals, mostly affecting carnivorous species (*4*) and massive die-off events raised concern about potential mammal-to-mammal transmission in dense populations (*5**,**6*). HPAIV infections were regularly characterized by high viral loads in the brain and associated clinical signs of the central nervous system with corresponding morphologic changes (*7**–**11*). Although Germany has had high HPAIV infection rates in avian species, prevalence studies on HPAIV infections in terrestrial predators, which feed on (infected) waterfowl, are not available. We performed a 1 year-survey to detect HPAIV in wild terrestrial predators in northern Germany.

## The Study

We studied HPAIV infections in 170 wildlife predators of several animal species: red foxes (*Vulpes vulpes*, n = 110), racoons (*Procyon lotor,* n = 28), badgers (*Meles meles,* n = 15), martens (*Martes foina* or *Martes martes*, n = 9), and racoon dogs (*Nyctereutes procyonoides,* n = 8) by using PCR and in situ methods. We performed avian influenza virus real-time PCR on individual brain samples of those 170 terrestrial wildlife predators and an H5-specific assay. The carcasses originated from different geographic locations in the German federal state of Lower Saxony and were delivered to the Lower Saxony State Office for Consumer Protection and Food Safety during February 2022–April 2023 (Appendix Table 1).

We detected viral RNA with cycle threshold values of 13.75–36.35 in the brains of 5 red foxes (4.5%), which were submitted with differing preliminary reports partly involving signs of disease specific for the central nervous system. Virus-positive animals were found in the first 3 months of 2023 (Table). In 4 of 5 cases, full-length influenza virus sequences could be recovered by using Illumina (https://www.illumina.com) high-throughput sequencing and were deposited in GISAID (https://gisaid.org) (Appendix Table 2). The hemagglutinin sequences of the 4 red foxes had nucleotide identities of 98.53%–99.06% and had a cleavage site typical for a highly pathogenic phenotype (REKRRKRG). 

**Table:** Overview of case history, qRT-PCR results, nucleotide frequency at amino acid position 627 of the PB2 segment, and virus isolation experiments of HPAIV H5N1-positive foxes, Germany*

Animal no.	Case history, date of submission	Ct values, M segment/H5 segment			PB2 mutation E627K		Virus isolation
					nt 1879 →G, %	nt 1879 →A, %	
		Brain stem	Cerebellum	Rhinencephalon			
51023-60	Adult, clinical signs, nonspecific, 2023 Jan	13.75/19.49	20.29/25.05	18.07/21.78	99.54	0.46	Positive
51023-61	Found dead, 2023 Jan	28.98/34.60	29.95/36.35	30.45/34.36	ND	ND	ND
51023-113	Found dead, 2023 Jan	29.16./32.32	31.02/35.55	29.05/32.51	4.53	95.47	ND
51023-124	Adult, clinical signs, CNS, 2023 Feb	23.16/27.61	25.91/30.04	25.40/29.77	99.93	0.07	Positive
51023-261	Pup, clinical signs, CNS, 2023 Mar	16.90/19.17	21.80/23.72	21.01/23.38	99.76	0.23	Positive

*CNS, central nervous system; H, hemagglutinin; HPAIV, highly pathogenic avian influenza virus; M, matrix; ND, not determined; nt, nucleotide; PB2, polymerase basic 2; qRT-PCR, quantitative reverse transcription PCR.

**Figure 1:**
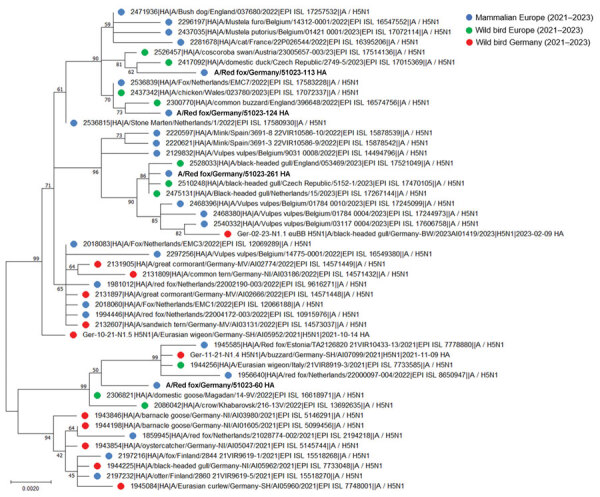
Phylogenetic analyses of highly pathogenic avian influenza virus H5 sequences of mammals and wild birds, Germany. The maximum-likelihood tree was built with 500 bootstrap iterations. H5 variants included 4 red foxes from Lower Saxony, Germany, their 3 closest relatives according to BLAST (https://blast.ncbi.nlm.nih.gov/Blast.cgi) analyses, and distinct H5 sequences detected in wild birds from northern Germany and in mammalian species from Europe during 2021–2023. Bold text indicates virus variants found in this study. Numbers along branches indicate percentage bootstrap values. Scale bar indicates nucleotide substitutions per site. H, hemagglutinin.

For the phylogenetic analyses, we included avian influenza virus nucleotide sequences representing the first 3 BLAST (https://blast.ncbi.nlm.nih.gov/Blast.cgi) search hits, as well as sequences detected in mammals during 2021‒2023 in Europe and relevant HPAIV H5N1 sequences recovered from wild birds from northern Germany. The phylogenetic tree showed that the H5 sequences detected in red foxes in this study belonged to clade 2.3.4.4b. They were closely related to sequences previously found in avian and mammal hosts in Europe but did not form a separate cluster (Figure 1). Analyses of all 8 genome segments assigned the genomes to genotype Ger-10–21-N1.5, Ger-11-21-N1.4, of Ger-02-23-N1.1 (Appendix Figure) (*12*). We further analyzed viral RNA segments for the presence of mutations, which have been described in mammalian H5Nx infections, involving amino acid residues in the polymerase basic (PB) 2 segment (*7*,*9*,*11*). The PB2 E627K substitution was found in the consensus sequence in only 1 of 4 cases (animal no. 51023–113) (Table).

HPAIV H5N1 isolates were obtained from 3 of 4 central nervous system tissue homogenates during the first passage in MDCK II cells. Hemagglutinating activity and high viral loads (cycle threshold values <20) were evident in the supernatant of inoculated cell cultures. An influenza-like cytopathic effect proceeded to affect the whole cell monolayer within less than 72 hours postinoculation (Table).

**Figure 2:**
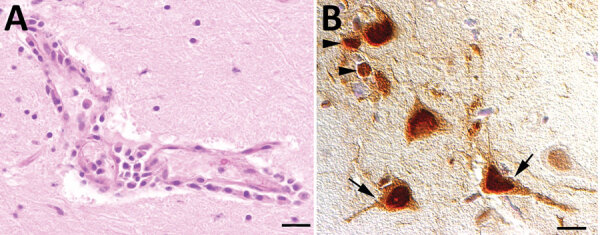
Results of testing for highly pathogenic avian influenza virus in red foxes (*Vulpes vulpes*), Germany. A) Microscopic findings in the brain of an influenza A(H5N1) virus‒infected red fox showing a lymphocytic to histiocytic perivascular encephalitis (hematoxylin and eosin stained, scale bar = 50 μm). B) Immunohistochemical demonstration of influenza A virus nucleoprotein in neurons (arrows) and glial cells (arrowheads) in the cerebrum of a virus‒infected red fox (a*vidin biotin complex* method, scale bar = 20 μm).

None of the 5 AIV-positive foxes showed macroscopic brain lesions. Microscopically, we observed mild-to-moderate multifocal lymphohistiocytic encephalitis with predominant perivascular infiltrations in the midbrain of 4 foxes and in the brain stem of 3 foxes. One animal showed vasculitis. Minor lesions were seen in the rhinencephalon and the cerebellum in 2 foxes. Neuronal necrosis was present in the midbrain and rhinencephalon in 1 animal, whereas another animal showed multifocal gliosis in the brain stem and midbrain. Immunohistologic analysis showed influenza A nucleoprotein in morphologically affected and unaffected areas of the brain. This protein was located in the nucleus and perikaryon and cell processes of neurons, as well as in glial cells (Figure 2).

## Conclusions

Recent H5N1 virus infections with dramatic losses in sea bird breeding colonies in Europe have proven the deleterious implications of a year-round presence of HPAIV in northern Europe (*13*). The sustained occurrence of HPAIV outbreaks in wild birds might enhance spillover risks to wild carnivores that prey on infected birds or scavenge on their carcasses. An alimentary route of infection has been proven experimentally (*14*).

Our phylogenetic analyses confirm that virus strains similar to those circulating in the wild bird population have the potential to be transferred to terrestrial carnivores. Wild birds are suspected to be the most likely animal reservoirs sustaining HPAIV replication in Europe. The role of other animal species, in particular mammalian predators, is still equivocal. Clinically conspicuous cases have been reported throughout Europe in a sporadic fashion, but the true prevalence remains unknown. Our data of a 1-year survey from the federal state Lower Saxony in Germany showed a temporal clustering of cases in red foxes found within the first 3 months of 2023. This period coincided with a peak in HPAIV detections in wild birds in northern Europe (*4*). Studies from the Netherlands also described positive cases in the winter period of 2021‒2022 (*10*,*11*). Conditions in the cold season could favor virus transmission to carnivorous mammals.

Virus variants from foxes in Germany did not form a separate phylogenetic cluster confirming independent infection events. According to several reports, gs/GD-like HPAIV shows a strong neurotropism in mammal species. This finding is true for H5N8 infections in harbor seals (*9*), as well as for disease outbreaks in terrestrial predators (*7**,**8**,**10**,**11*). Also, in this study, HPAIV infection of the brain was shown by high viral loads and immunohistochemical analysis. In previous studies, point mutations suspected to increase viral replication in mammals have been frequently described, especially the E627K mutation in the PB2 segment. Ten of 14 HPAIV H5N1-positive wild carnivores detected in the Netherlands carried the mammal-adaptive variant (*10**,**11*), and those viruses replicated to higher titers in mammalian cells than in an avian cell line (*10*). In our study, only 1 of 4 four analyzed cases had the E627K substitution. Those results support previous observations that PB2 627K is not a prerequisite for virus replication in mammalian cells (*10*).

Little is known about the pathogenesis of HPAIV infection in wild mammal predators. Further virologic and serologic studies are planned and are needed to monitor those potential hosts as indicators for enhanced zoonotic spillover. Recent serologic findings of a clinically silent HPAIV H5N1 infection in a pig herd in Italy suggest that the neurologic cases seen in carnivores in Europe and elsewhere might represent just the tip of an iceberg (*15*). Widespread HPAIV H5N1 infection in those hosts would provide ample opportunities to further adapt to mammals, which could be associated with increased infection risks for humans.

Appendix Additional information on neurotropic highly pathogenic avian influenza virus H5N1 in red foxes, northern Germany
