# Supplementary material for: Neurotropic Highly Pathogenic Avian Influenza A(H5N1) Virus in Red Foxes, Northern Germany
Source: Emerg Infect Dis. 2023 Dec;29(12):2509–12. doi: 10.3201/eid2912.230938 (PMC10683810; doi:10.3201/eid2912.230938)
Supplement: Appendix — Additional information on neurotropic highly pathogenic avian influenza virus H5N1 in red foxes, northern Germany [file 23-0938-Techapp-s1.pdf]

Article DOI: https://doi.org/10.3201/eid2912.230938

EID cannot ensure accessibility for Supplemental Materials supplied by authors. Readers who have difficulty accessing supplementary content should contact the authors for assistance.

Neurotropic Highly Pathogenic Avian Influenza A(H5N1) Virus in Red Foxes, Northern Germany

Appendix

Materials and Methods

RNA extraction, PCR investigations and Next Generation Sequencing (NGS) analyses

Viral RNA was extracted from the brain samples with the NucleoMag VET Kit (Macherey-Nagel) on a KingFisher 96-Flex machine, and real-time PCR targeting the viral M segment was performed with a commercial kit (Virotype Influenza A RT-PCR Kit, Indical Bioscience) according to the manufacturer´s instructions. A second real-time PCR was used to confirm the H5 subtype in the samples with the following primers and probe (AIV-H5LH1: 5‘-ACA TAT GAC TAC CCA CAR TAT TCA G-3‘, AIV-H5RH1: 5`-AGA CCA GCT AYC ATG ATT GC-3‘, 5`-FAM-TCA ACA GTG GCG AGT TCC CTA GCA-TAMRA-3‘) under these conditions: 50°C, 30min; 94°C, 2min; 40 × (94°C, 30sec; 56°C, 30sec; 68°C, 30sec) by using the SuperScript III One-Step RT-RCR-Kit (Invitrogen).

To recover full-length viral genome sequences, viral RNA segments were reverse transcribed and amplified with the Invitrogen Superscript III One-Step RT-PCR Kit (Thermo Fisher Scientific) with a universal Primer Set targeting conserved regions of all eight segments (*1*). Primers were adjusted to a stock concentration of 10pmol/µl. The RT-PCR reaction consisted of 12.5µl 2x reaction buffer, 1µl of each primer, 1µl of Superscript III RT / Platinum Taq enzyme mix, 3.5µl nuclease free water and 5µl RNA sample adding up to a total volume of 25µl. The RT-PCR cycler program consisted of an initial 45-minute period at 45°C followed by a 2-minute denaturation step at 94°C, then 5 cycles of 94°C for 30s, 45°C for 30s and 68°C for 3 minutes followed by 30 cycles of 94°C for 30s, 57°C for 30s and 68°C for 3 minutes. The final Elongation step was 68°C for 5 minutes.

Following this, Illumina compatible DNA libraries were prepared (ILMN DNA LP (M) Tagmentation Kit) and sequenced on an Illumina NextSeq 1000 benchtop sequencer. The quality of the reads was checked with FastQC (*2*) and fastp (*3*). The final AIV genome assembly was performed with IRMA (*4*).

Histopathological and immunohistochemical examination

Samples were collected from different organs and tissues including rhinencephalon, the cerebellum and other parts of the brain, fixed in 10% neutral buffered formalin, processed by routine methods, embedded in paraffin wax, sectioned (4µm), and stained with hematoxylin and eosin (H&E).

Immunohistochemistry was performed on paraffin-embedded tissue sections by using a murine monoclonal antibody specific for the nucleoprotein (*5*). Briefly, 3µm tissue sections were deparaffinized and rehydrated using a descending series of alcohol. Afterwards, slides were incubated in 85% ethanol with additional 0.5% H_2O_2 for 30 min. at room temperature (RT) for blocking endogenous peroxidase activities. Enzymatic pre-treatment with proteinase K was performed for 10min. at RT and unspecific background staining was blocked with inactivated goat serum diluted 1:5 in phosphate-buffered saline (PBS). The primary anti-influenza A antibody (CLONEGENE LLC, HB65) was diluted 1:200 in PBS with 1% bovine serum albumin and slides were incubated overnight at 4°C. For negative control, the primary antibody was substituted by ascites fluid from Balb/c mice. Biotinylated goat-anti-mouse IgG was used as secondary antibody and added for 45min at RT. For visualization of immunostaining, the avidin-biotin-peroxidase complex (VECTASTAIN Elite ABC Kit; Vector Laboratories, Burlingame, CA) was applied by using 3,3`-diaminobenzidine-tetrahydrochloride (Sigma Aldrich) as chromogen. Counterstaining was performed with Mayer`s hemalaun.

References

1. Hoffmann E, Stech J, Guan Y, Webster RG, Perez DR. Universal primer set for the full-length amplification of all influenza A viruses. Arch Virol. 2001;146:2275–89 . PubMed https://doi.org/10.1007/s007050170002

2. Andrews S. 2010. FASTQC. A quality control tool for high throughput sequence data [cited 2023 Oct 4].http://www.bioinformatics.babraham.ac.uk/projects/fastqc

3. Chen S, Zhou Y, Chen Y, Gu J. fastp: an ultra-fast all-in-one FASTQ preprocessor. Bioinformatics. 2018;34:i884–90. PubMed https://doi.org/10.1093/bioinformatics/bty560

4. Shepard SS, Meno S, Bahl J, Wilson MM, Barnes J, Neuhaus E. Viral deep sequencing needs an adaptive approach: IRMA, the iterative refinement meta-assembler. BMC Genomics. 2016;17:708. PubMed https://doi.org/10.1186/s12864-016-3030-6

5. de Boer GF, Back W, Osterhaus AD. An ELISA for detection of antibodies against influenza A nucleoprotein in humans and various animal species. Arch Virol. 1990;115:47–61. PubMed https://doi.org/10.1007/BF01310622

Appendix Table 1. Overview of wild terrestrial carnivores examined from February 2022 to April 2023. The months in which animals tested positive are in bold type*

	Number of animals tested/number of AIV positive animals					
Year/month	Red fox	Racoon	Badger	Marten	Racoon dog	Total
2022/Feb	11/0	2/0	3/0	0/0	1/0	17/0
2022/Mar	17/0	2/0	0/0	1/0	0/0	20/0
2022/Apr	2/0	0/0	0/0	1/0	0/0	3/0
2022/May	7/0	0/0	1/0	0/0	0/0	8/0
2022/Jun	6/0	0/0	0/0	1/0	0/0	7/0
2022/Jul	4/0	3/0	2/0	1/0	0/0	10/0
2022/Aug	3/0	1/0	2/0	0/0	1/0	7/0
2022/Sep	4/0	3/0	0/0	0/0	0/0	7/0
2022/Oct	8/0	9/0	2/0	0/0	1/0	20/0
2022/Nov	5/0	1/0	3/0	0/0	2/0	11/0
2022/Dec	10/0	5/0	0/0	1/0	1/0	17/0
**2023/Jan**	**9/3**	0/0	2/0	0/0	0/0	**11/3**
**2023/Feb**	**9/1**	0/0	0/0	1/0	2/0	**12/1**
**2023/Mar**	**12/1**	1/0	0/0	2/0	0/0	**15/1**
2023/Apr	3/0	1/0	0/0	1/0	0/0	5/0
Total	110/5	28/0	15/0	9/0	8/0	170/5

*Months in which animals tested positive are in bold type.

Appendix Table 2. Isolates used in this study*

Isolate ID	Country	Collection date	Isolate name	Originating Laoratory	Submitting Laboratory	Authors
EPI_ISL_17521049	United Kingdom	2023-Mar-23	A/black-headed_gull/England/053469/2023	Animal and Plant Health Agency (APHA)	Animal and Plant Health Agency (APHA)	
EPI_ISL_17470105	Czech Republic	2023-Apr-04	A/black-headed_gull/Czech_Republic/5152–1/2023	State Veterinary Institute Prague	State Veterinary Institute Prague	Alexander,Nagy;Lenka,Cernikova;Martina,Stara
EPI_ISL_17267144	Netherlands	2023-Mar-01	A/Black-headed gull/Netherlands/15/2023	Erasmus Medical Center	Erasmus Medical Center	
EPI_ISL_17606758	Belgium	2023-Mar-29	A/Vulpes_vulpes/Belgium/03117_0004/2023	Sciensano - Animal Infectious Diseases	Sciensano, Department of Animal Infectious Diseases	Van Borm, Steven; Roupie, Virginie; Hostyn, Pierre; Mathijs, Elisabeth; Lambrecht, Benedicte; Steensels, Mieke
EPI_ISL_17245099	Belgium	2023-Feb-27	A/Vulpes_vulpes/Belgium/01784_0010/2023	Sciensano - Animal Infectious Diseases	Sciensano, Department of Animal Infectious Diseases	Van Borm, Steven; Roupie, Virginie; Hostyn, Pierre; Mathijs, Elisabeth; Lambrecht, Benedicte; Steensels, Mieke
EPI_ISL_17244973	Belgium	2023-Feb-27	A/Vulpes_vulpes/Belgium/01784_0004/2023	Sciensano - Animal Infectious Diseases	Sciensano, Department of Animal Infectious Diseases	Van Borm, Steven; Roupie, Virginie; Hostyn, Pierre; Mathijs, Elisabeth; Lambrecht, Benedicte; Steensels, Mieke
EPI_ISL_14494796	Belgium	2022-Jun-16	A/Vulpes_vulpes/Belgium/9031_0008/2022	Sciensano - Animal Infectious Diseases	Sciensano, Department of Animal Infectious Diseases	Van Borm, Steven; Mathijs, Elisabeth; Roupie, Virginie; Vervaeke, Muriel; Lambrecht, Benedicte; Steensels, Mieke
EPI_ISL_15878539	Spain	2022-Oct-18	A/Mink/Spain/3691–8_22VIR10586–10/2022	Laboratorio Central de Veterinaria	Istituto Zooprofilattico Sperimentale delle Venezie	Ruano, M.J.; Rocha, A.; Sanchez, A.; Aguero, M; Barbierato, G.; Zecchin, B.; Fusaro, A.; Schivo, A.; Salviato, A.; Palumbo, E.; Giussani, E.; Pastori, A.; Monne, I.; Terregino, C.
EPI_ISL_15878542	Spain	2022-Oct-18	A/Mink/Spain/3691–3_22VIR10586–9/2022	Laboratorio Central de Veterinaria	Istituto Zooprofilattico Sperimentale delle Venezie	Ruano, M.J.; Rocha, A.; Sanchez, A.; Aguero, M; Barbierato, G.; Zecchin, B.; Fusaro, A.; Schivo, A.; Salviato, A.; Palumbo, E.; Giussani, E.; Pastori, A.; Monne, I.; Terregino, C.
EPI_ISL_12069289	Netherlands	2022-Jan-22	A/Fox/Netherlands/EMC3/2022	Erasmus Medical Center	Erasmus Medical Center	R.A.M., Fouchier; S., Thewessen; O., Vuong; I., Chestakova; R., Sikkema; P., Lexmond; M., Pronk
EPI_ISL_14571448	Germany	2022-May-03	A/great cormorant/Germany-MV/AI02666/2022	Landesamt für Landwirtschaft, Lebensmittelsicherheit und Fischerei (LALLF)	Friedrich-Loeffler-Institut	
EPI_ISL_14573037	Germany	2022-May-31	A/sandwich tern/Germany-MV/AI03131/2022	Landesamt für Landwirtschaft, Lebensmittelsicherheit und Fischerei (LALLF)	Friedrich-Loeffler-Institut	

Isolate ID	Country	Collection date	Isolate name	Originating Laoratory	Submitting Laboratory	Authors
EPI_ISL_16549380	Belgium	2022-Dec-08	A/Vulpes_vulpes/Belgium/14775–0001/2022	Sciensano - Animal Infectious Diseases	Sciensano, Department of Animal Infectious Diseases	Van Borm, Steven; Roupie, Virginie; Hostyn, Pierre; Lambrecht, Benedicte; Steensels, Mieke
EPI_ISL_12066188	Netherlands	2022-Jan-26	A/Fox/Netherlands/EMC1/2022	Erasmus Medical Center	Erasmus Medical Center	R.A.M., Fouchier; S., Therwessen; O., Vuong; I., Chestakova; R., Sikkema; P., Lexmond; M., Pronk
EPI_ISL_9616271	Netherlands	2022-Jan-31	A/red fox/Netherlands/22002190–003/2022	Wageningen Bioveterinary Research	Wageningen Bioveterinary Research	Beerens, Nancy; Harders, Frank; Pritz-Verschuren, Sylvia; Roose, Marit; Venema, Sandra; Germeraad, Evelien; Engelsma, Marc; Heutink, Rene
EPI_ISL_10915976	Netherlands	2022-Mar-01	A/red fox/Netherlands/22004172–003/2022	Wageningen Bioveterinary Research	Wageningen Bioveterinary Research	Beerens, Nancy; Harders, Frank; Pritz-Verschuren, Sylvia; Roose, Marit; Venema, Sandra; Germeraad, Evelien; Engelsma, Marc; Heutink, Rene
EPI_ISL_14571449	Germany	2022-May-06	A/great cormorant/Germany-MV/AI02774/2022	Landesamt für Landwirtschaft, Lebensmittelsicherheit und Fischerei (LALLF)	Friedrich-Loeffler-Institut	
EPI_ISL_14571432	Germany	2022-Jun-07	A/common tern/Germany-NI/AI03186/2022	Lebensmittel- und Veterinärinstitut Oldenburg - Standort Veterinärinstitut	Friedrich-Loeffler-Institut	
EPI_ISL_17580930	Netherlands	2022-Jan-16	A/Stone Marten/Netherlands/1/2022	Erasmus Medical Center	Erasmus Medical Center	Vuong,O;Thewessen, S;Bellido-Martin,A.B; Chestakova, I; Sikkema, R;Fouchier, R.A.M.
EPI_ISL_17583228	Netherlands	2022-Sep-13	A/Fox/Netherlands/EMC7/2022	Erasmus Medical Center	Erasmus Medical Center	Vuong,O;Thewessen, S;Bellido-Martin,A.B; Chestakova, I; Sikkema, R;Fouchier, R.A.M.
EPI_ISL_17072337	United Kingdom	2023-Feb-11	A/chicken/Wales/023780/2023	Animal and Plant Health Agency (APHA)	Animal and Plant Health Agency (APHA)	
EPI_ISL_16574756	United Kingdom	2022-Dec-20	A/common_buzzard/England/396648/2022	Animal and Plant Health Agency (APHA)	Animal and Plant Health Agency (APHA)	
EPI_ISL_17015369	Czech Republic	2023-Feb-16	A/domestic_duck/Czech_Republic/2749–5/2023	State Veterinary Institute Prague	State Veterinary Institute Prague	Alexander,Nagy;Lenka,Cernikova;Martina,Stara
EPI_ISL_17514136	Austria	2023-Jan-18	A/coscoroba swan/Austria/23005657–003/23	Institute for Veterinary Disease Control Moedling, Austrian Agency for Health and Food Safety	Austrian Agency for Health and Food Safety (AGES)	

Isolate ID	Country	Collection date	Isolate name	Originating Laoratory	Submitting Laboratory	Authors
EPI_ISL_16395206	France	2022-Dec-24	A/cat/France/22P026544/2022	Anses (Ploufragan-Plouzané)	ANSES Agence Nationale De Securite Sanitaire De L'alimentation	
EPI_ISL_17257532	United Kingdom	2022-Nov-18	A/Bush_dog/England/037680/2022	Animal and Plant Health Agency (APHA)	Animal and Plant Health Agency (APHA)	
EPI_ISL_16547552	Belgium	2022-Dec-08	A/Mustela_furo/Belgium/14312–0001/2022	Sciensano - Animal Infectious Diseases	Sciensano, Department of Animal Infectious Diseases	Van Borm, Steven; Roupie, Virginie; Hostyn, Pierre; Lambrecht, Benedicte; Steensels, Mieke
EPI_ISL_17072114	Belgium	2023-Feb-09	A/Mustela_putorius/Belgium/01421_0001/2023	Sciensano - Animal Infectious Diseases	Sciensano, Department of Animal Infectious Diseases	Van Borm, Steven; Roupie, Virginie; Hostyn, Pierre; Mathijs, Elisabeth; Lambrecht, Benedicte; Steensels, Mieke
EPI_ISL_7778880	Estonia	2021-Nov-08	A/Red_fox/Estonia/TA2126820_21VIR10433–13/2021	Estonian Veterinary and Food Laboratory	Istituto Zooprofilattico Sperimentale Delle Venezie	Nurmoja, I.; Vilem, A.; Juurik, T.; Zecchin, B.; Fusaro, A.; Schivo, A.; Salviato, A.; Palumbo, E.; Milani, A.; Giussani, E.; Pastori, A.; Monne, I.; Terregino, C.
EPI_ISL_11725994	Germany	2021-Nov-09	A/buzzard/Germany-SH/AI07099/2021	Landeslabor Schleswig-Holstein	Friedrich-Loeffler-Institut	
EPI_ISL_8650947	Netherlands	2022-Jan-03	A/red fox/Netherlands/22000097–004/2022	Wageningen Bioveterinary Research	Wageningen Bioveterinary Research	Beerens, Nancy; Harders, Frank; Pritz-Verschuren, Sylvia; Roose, Marit; Venema, Sandra; Germeraad, Evelien; Engelsma, Marc; Heutink, Rene
EPI_ISL_16618971	Russian Federation	2022-Oct-12	A/domestic goose/Magadan/14–9V/2022	State Research Center of Virology and Biotechnology (VECTOR)	State Research Center of Virology and Biotechnology (VECTOR)	
EPI_ISL_13692635	Russian Federation	2022-Apr-21	A/crow/Khabarovsk/216–13V/2022	State Research Center of Virology and Biotechnology (VECTOR)	State Research Center of Virology and Biotechnology (VECTOR)	
EPI_ISL_5146291	Germany	2021-Apr-14	A/barnacle goose/Germany-NI/AI03980/2021	Lebensmittel- und Veterinärinstitut Oldenburg - Standort Veterinärinstitut	Friedrich-Loeffler-Institut	
EPI_ISL_2194218	Netherlands	2021-May-17	A/red fox/Netherlands/21028774–002/2021	Wageningen Bioveterinary Research	Wageningen Bioveterinary Research	Beerens, Nancy; Harders, Frank; Pritz-Verschuren, Sylvia; Roose, Marit; Germeraad, Evelien; Engelsma, Marc; Bossers, Alex; Heutink, Rene
EPI_ISL_5099456	Germany	2021-Feb-17	A/barnacle goose/Germany-NI/AI01605/2021	Lebensmittel- und Veterinärinstitut Oldenburg - Standort Veterinärinstitut	Friedrich-Loeffler-Institut	

Isolate ID	Country	Collection date	Isolate name	Originating Laoratory	Submitting Laboratory	Authors
EPI_ISL_5145744	Germany	2021-Jul-17	A/oystercatcher/Germany-NI/AI05047/2021	Lebensmittel- und Veterinärinstitut Oldenburg - Standort Veterinärinstitut	Friedrich-Loeffler-Institut	
EPI_ISL_15518268	Finland	2021-Sep-01	A/fox/Finland/2844_21VIR9619–1/2021	Finnish Food Authority	Istituto Zooprofilattico Sperimentale Delle Venezie	Tammiranta, N.;Kantala, T.;Laamanen, I.;Gadd, T.;Zecchin, B.;Fusaro, A.;Schivo, A.;Salviato, A.;Palumbo, E.;Giussani, E.;Pastori, A.;Monne, I.;Terregino, C.
EPI_ISL_7733048	Germany	2021-Oct-12	A/black-headed gull/Germany-NI/AI05962/2021	Lebensmittel- und Veterinärinstitut Oldenburg - Standort Veterinärinstitut	Friedrich-Loeffler-Institut	
EPI_ISL_15518270	Finland	2021-Sep-01	A/otter/Finland/2860_21VIR9619–5/2021	Finnish Food Authority	Istituto Zooprofilattico Sperimentale Delle Venezie	Tammiranta, N.;Kantala, T.;Laamanen, I.;Gadd, T.;Zecchin, B.;Fusaro, A.;Schivo, A.;Salviato, A.;Palumbo, E.;Milani, A.;Giussani, E.;Pastori, A.;Monne, I.;Terregino, C.
EPI_ISL_7748001	Germany	2021-Oct-14	A/Eurasian curlew/Germany-SH/AI05960/2021	Landeslabor Schleswig-Holstein	Friedrich-Loeffler-Institut	
EPI_ISL_7733585	Italy	2021-Oct-28	A/Eurasian_wigeon/Italy/21VIR8919–3/2021	Istituto Zooprofilattico Speriment. Lombardia, Emilia Ro. IZSLER	Istituto Zooprofilattico Sperimentale Delle Venezie	Zecchin, B.; Fusaro, A.; Schivo, A.; Salviato, A.; Palumbo, E.; Milani, A.; Giussani, E.; Pastori, A.; Monne, I.; Terregino, C.
EPI_ISL_17880904	Germany	2023-Jan-09	A/Red fox/Germany/51023–60/2023	Lower Saxony State Office for Consumer Protection and Food Safety	Lower Saxony State Office for Consumer Protection and Food Safety	This study
EPI_ISL_17880908	Germany	2023-Jan-31	A/Red fox/Germany/51023–113/2023	Lower Saxony State Office for Consumer Protection and Food Safety	Lower Saxony State Office for Consumer Protection and Food Safety	This study
EPI_ISL_17880911	Germany	2023-Feb-09	A/Red fox/Germany/51023–124/2023	Lower Saxony State Office for Consumer Protection and Food Safety	Lower Saxony State Office for Consumer Protection and Food Safety	This study
EPI_ISL_17880914	Germany	2023-Mar-29	A/Red fox/Germany/51023–261/2023	Lower Saxony State Office for Consumer Protection and Food Safety	Lower Saxony State Office for Consumer Protection and Food Safety	This study

*We gratefully acknowledge the authors, originating and submitting laboratories of the sequences from GISAID's EpiFlu Database on which this research is based. All submitters of data may be contacted directly via www.gisaid.org. Sequences obtained in this study are listed at the end of this table.

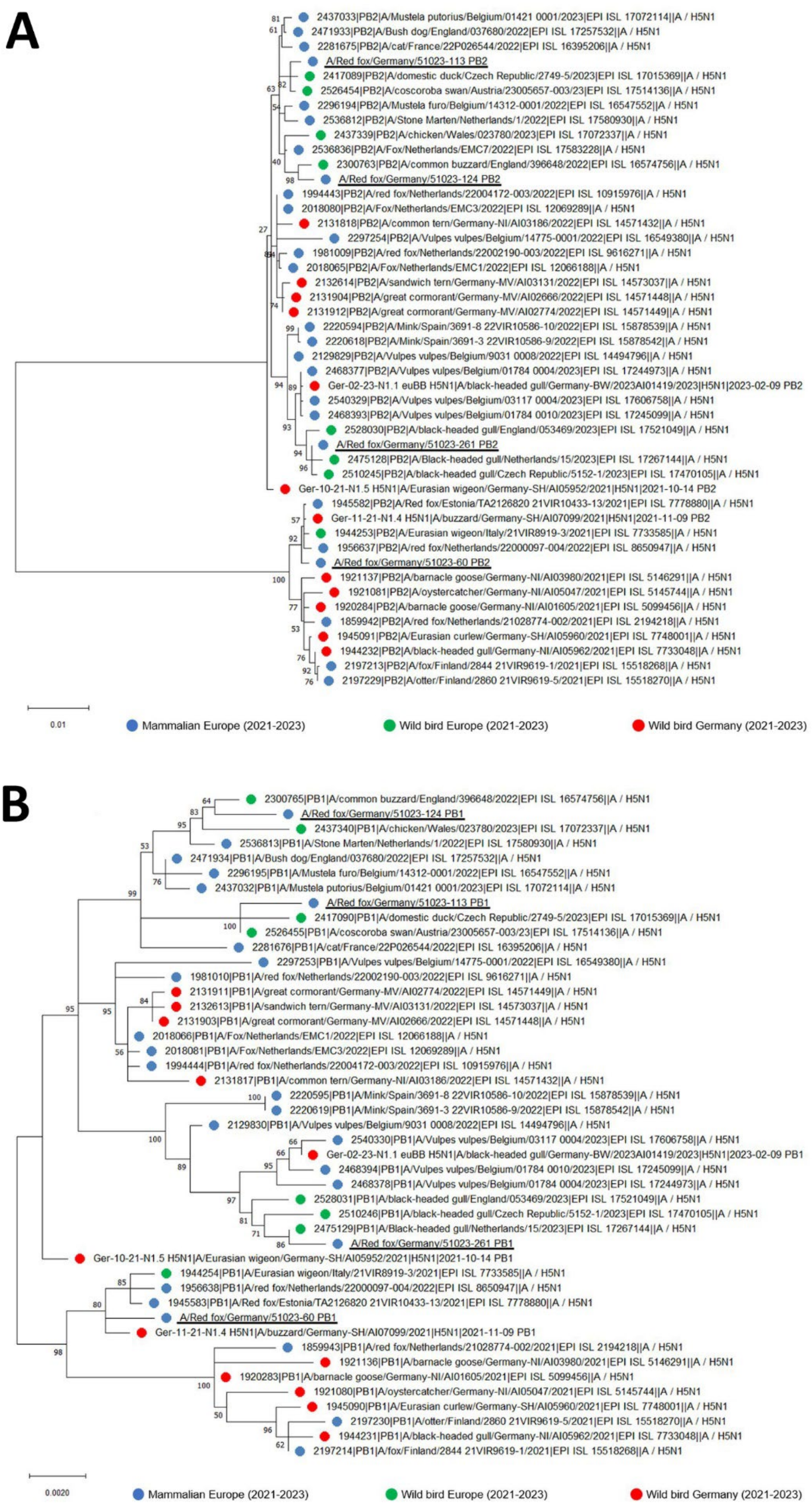
A
2437033|PB2|A/Mustela putorius/Belgium/01421 0001/2023|EPI ISL 17072114||A / H5N1
2471933|PB2|A/Bush dog/England/037680/2022|EPI ISL 17257532||A / H5N1
2281675|PB2|A/cat/France/22P026544/2022|EPI ISL 16395206||A / H5N1
A/Red fox/Germany/51023-113 PB2
2417089|PB2|A/domestic duck/Czech Republic/2749-5/2023|EPI ISL 17015369||A / H5N1
2526454|PB2|A/coscoroba swan/Austria/23005657-003/23|EPI ISL 17514136||A / H5N1
2296194|PB2|A/Mustela furo/Belgium/14312-0001/2022|EPI ISL 16547552||A / H5N1
2536812|PB2|A/Stone Marten/Netherlands/1/2022|EPI ISL 17580930||A / H5N1
2437339|PB2|A/chicken/Wales/023780/2023|EPI ISL 17072337||A / H5N1
2536836|PB2|A/Fox/Netherlands/EMC7/2022|EPI ISL 17583228||A / H5N1
2300763|PB2|A/common buzzard/England/396648/2022|EPI ISL 16574756||A / H5N1
A/Red fox/Germany/51023-124 PB2
1994443|PB2|A/red fox/Netherlands/22004172-003/2022|EPI ISL 10915976||A / H5N1
2018080|PB2|A/Fox/Netherlands/EMC3/2022|EPI ISL 12069289||A / H5N1
2131818|PB2|A/common tern/Germany-NI/AI03186/2022|EPI ISL 14571432||A / H5N1
2297254|PB2|A/Vulpes vulpes/Belgium/14775-0001/2022|EPI ISL 16549380||A / H5N1
1981009|PB2|A/red fox/Netherlands/22002190-003/2022|EPI ISL 9616271||A / H5N1
2018065|PB2|A/Fox/Netherlands/EMC1/2022|EPI ISL 12066188||A / H5N1
2132614|PB2|A/sandwich tern/Germany-MV/AI03131/2022|EPI ISL 14573037||A / H5N1
2131904|PB2|A/great cormorant/Germany-MV/AI02666/2022|EPI ISL 14571448||A / H5N1
2131912|PB2|A/great cormorant/Germany-MV/AI02774/2022|EPI ISL 14571449||A / H5N1
2220594|PB2|A/Mink/Spain/3691-8 22VIR10586-10/2022|EPI ISL 15878539||A / H5N1
2220618|PB2|A/Mink/Spain/3691-3 22VIR10586-9/2022|EPI ISL 15878542||A / H5N1
2129829|PB2|A/Vulpes vulpes/Belgium/9031 0008/2022|EPI ISL 14494796||A / H5N1
2468377|PB2|A/Vulpes vulpes/Belgium/01784 0004/2023|EPI ISL 17244973||A / H5N1
Ger-02-23-N1.1 euBB H5N1|A/black-headed gull/Germany-BW/2023AI01419/2023|H5N1|2023-02-09 PB2
2540329|PB2|A/Vulpes vulpes/Belgium/03117 0004/2023|EPI ISL 17606758||A / H5N1
2468393|PB2|A/Vulpes vulpes/Belgium/01784 0010/2023|EPI ISL 17245099||A / H5N1
2528030|PB2|A/black-headed gull/England/053469/2023|EPI ISL 17521049||A / H5N1
A/Red fox/Germany/51023-261 PB2
2475128|PB2|A/Black-headed gull/Netherlands/15/2023|EPI ISL 17267144||A / H5N1
2510245|PB2|A/black-headed gull/Czech Republic/5152-1/2023|EPI ISL 17470105||A / H5N1
Ger-10-21-N1.5 H5N1|A/Eurasian wigeon/Germany-SH/AI05952/2021|H5N1|2021-10-14 PB2
1945582|PB2|A/Red fox/Estonia/TA2126820 21VIR10433-13/2021|EPI ISL 7778880||A / H5N1
Ger-11-21-N1.4 H5N1|A/buzzard/Germany-SH/AI07099/2021|H5N1|2021-11-09 PB2
1944253|PB2|A/Eurasian wigeon/Italy/21VIR8919-3/2021|EPI ISL 7733585||A / H5N1
1956637|PB2|A/red fox/Netherlands/22000097-004/2022|EPI ISL 8650947||A / H5N1
A/Red fox/Germany/51023-60 PB2
1921137|PB2|A/barnacle goose/Germany-NI/AI03980/2021|EPI ISL 5146291||A / H5N1
1921081|PB2|A/oystercatcher/Germany-NI/AI05047/2021|EPI ISL 5145744||A / H5N1
1920284|PB2|A/barnacle goose/Germany-NI/AI01605/2021|EPI ISL 5099456||A / H5N1
1859942|PB2|A/red fox/Netherlands/21028774-002/2021|EPI ISL 2194218||A / H5N1
1945091|PB2|A/Eurasian curlew/Germany-SH/AI05960/2021|EPI ISL 7748001||A / H5N1
1944232|PB2|A/black-headed gull/Germany-NI/AI05962/2021|EPI ISL 7733048||A / H5N1
2197213|PB2|A/fox/Finland/2844 21VIR9619-1/2021|EPI ISL 15518268||A / H5N1
2197229|PB2|A/otter/Finland/2860 21VIR9619-5/2021|EPI ISL 15518270||A / H5N1
0.01
Mammalian Europe (2021-2023)
Wild bird Europe (2021-2023)
Wild bird Germany (2021-2023)
B
2300765|PB1|A/common buzzard/England/396648/2022|EPI ISL 16574756||A / H5N1
A/Red fox/Germany/51023-124 PB1
2437340|PB1|A/chicken/Wales/023780/2023|EPI ISL 17072337||A / H5N1
2536813|PB1|A/Stone Marten/Netherlands/1/2022|EPI ISL 17580930||A / H5N1
2471934|PB1|A/Bush dog/England/037680/2022|EPI ISL 17257532||A / H5N1
2296195|PB1|A/Mustela furo/Belgium/14312-0001/2022|EPI ISL 16547552||A / H5N1
2437032|PB1|A/Mustela putorius/Belgium/01421 0001/2023|EPI ISL 17072114||A / H5N1
A/Red fox/Germany/51023-113 PB1
2417090|PB1|A/domestic duck/Czech Republic/2749-5/2023|EPI ISL 17015369||A / H5N1
2526455|PB1|A/coscoroba swan/Austria/23005657-003/23|EPI ISL 17514136||A / H5N1
2281676|PB1|A/cat/France/22P026544/2022|EPI ISL 16395206||A / H5N1
2297253|PB1|A/Vulpes vulpes/Belgium/14775-0001/2022|EPI ISL 16549380||A / H5N1
1981010|PB1|A/red fox/Netherlands/22002190-003/2022|EPI ISL 9616271||A / H5N1
2131911|PB1|A/great cormorant/Germany-MV/AI02774/2022|EPI ISL 14571449||A / H5N1
2132613|PB1|A/sandwich tern/Germany-MV/AI03131/2022|EPI ISL 14573037||A / H5N1
2131903|PB1|A/great cormorant/Germany-MV/AI02666/2022|EPI ISL 14571448||A / H5N1
2018066|PB1|A/Fox/Netherlands/EMC1/2022|EPI ISL 12066188||A / H5N1
2018081|PB1|A/Fox/Netherlands/EMC3/2022|EPI ISL 12069289||A / H5N1
1994444|PB1|A/red fox/Netherlands/22004172-003/2022|EPI ISL 10915976||A / H5N1
2131817|PB1|A/common tern/Germany-NI/AI03186/2022|EPI ISL 14571432||A / H5N1
2220595|PB1|A/Mink/Spain/3691-8 22VIR10586-10/2022|EPI ISL 15878539||A / H5N1
2220619|PB1|A/Mink/Spain/3691-3 22VIR10586-9/2022|EPI ISL 15878542||A / H5N1
2129830|PB1|A/Vulpes vulpes/Belgium/9031 0008/2022|EPI ISL 14494796||A / H5N1
2540330|PB1|A/Vulpes vulpes/Belgium/03117 0004/2023|EPI ISL 17606758||A / H5N1
Ger-02-23-N1.1 euBB H5N1|A/black-headed gull/Germany-BW/2023AI01419/2023|H5N1|2023-02-09 PB1
2468394|PB1|A/Vulpes vulpes/Belgium/01784 0010/2023|EPI ISL 17245099||A / H5N1
2468378|PB1|A/Vulpes vulpes/Belgium/01784 0004/2023|EPI ISL 17244973||A / H5N1
2528031|PB1|A/black-headed gull/England/053469/2023|EPI ISL 17521049||A / H5N1
2510246|PB1|A/black-headed gull/Czech Republic/5152-1/2023|EPI ISL 17470105||A / H5N1
2475129|PB1|A/Black-headed gull/Netherlands/15/2023|EPI ISL 17267144||A / H5N1
A/Red fox/Germany/51023-261 PB1
Ger-10-21-N1.5 H5N1|A/Eurasian wigeon/Germany-SH/AI05952/2021|H5N1|2021-10-14 PB1
1944254|PB1|A/Eurasian wigeon/Italy/21VIR8919-3/2021|EPI ISL 7733585||A / H5N1
1956638|PB1|A/red fox/Netherlands/22000097-004/2022|EPI ISL 8650947||A / H5N1
1945583|PB1|A/Red fox/Estonia/TA2126820 21VIR10433-13/2021|EPI ISL 7778880||A / H5N1
A/Red fox/Germany/51023-60 PB1
Ger-11-21-N1.4 H5N1|A/buzzard/Germany-SH/AI07099/2021|H5N1|2021-11-09 PB1
1859943|PB1|A/red fox/Netherlands/21028774-002/2021|EPI ISL 2194218||A / H5N1
1921136|PB1|A/barnacle goose/Germany-NI/AI03980/2021|EPI ISL 5146291||A / H5N1
1920283|PB1|A/barnacle goose/Germany-NI/AI01605/2021|EPI ISL 5099456||A / H5N1
1921080|PB1|A/oystercatcher/Germany-NI/AI05047/2021|EPI ISL 5145744||A / H5N1
1945090|PB1|A/Eurasian curlew/Germany-SH/AI05960/2021|EPI ISL 7748001||A / H5N1
2197230|PB1|A/otter/Finland/2860 21VIR9619-5/2021|EPI ISL 15518270||A / H5N1
1944231|PB1|A/black-headed gull/Germany-NI/AI05962/2021|EPI ISL 7733048||A / H5N1
2197214|PB1|A/fox/Finland/2844 21VIR9619-1/2021|EPI ISL 15518268||A / H5N1
0.0020
Mammalian Europe (2021-2023)
Wild bird Europe (2021-2023)
Wild bird Germany (2021-2023)

C

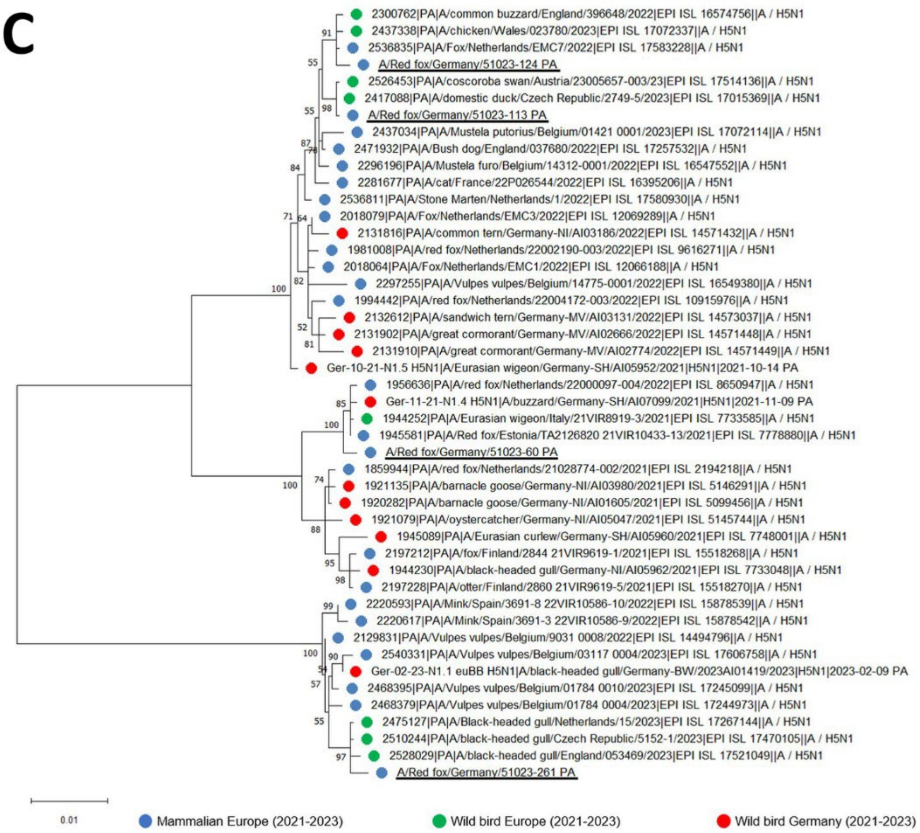
2300762|PA|A/common buzzard/England/396648/2022|EPI ISL 16574756||A / H5N1
2437338|PA|A/chicken/Wales/023780/2023|EPI ISL 17072337||A / H5N1
2536835|PA|A/Fox/Netherlands/EMC7/2022|EPI ISL 17583228||A / H5N1
A/Red fox/Germany/51023-124 PA
2526453|PA|A/coscoroba swan/Austria/23005657-003/23|EPI ISL 17514136||A / H5N1
2417088|PA|A/domestic duck/Czech Republic/2749-5/2023|EPI ISL 17015369||A / H5N1
A/Red fox/Germany/51023-113 PA
2437034|PA|A/Mustela putorius/Belgium/01421 0001/2023|EPI ISL 17072114||A / H5N1
2471932|PA|A/Bush dog/England/037680/2022|EPI ISL 17257532||A / H5N1
2296196|PA|A/Mustela furo/Belgium/14312-0001/2022|EPI ISL 16547552||A / H5N1
2281677|PA|A/cat/France/22P026544/2022|EPI ISL 16395206||A / H5N1
2536811|PA|A/Stone Marten/Netherlands/1/2022|EPI ISL 17580930||A / H5N1
2018079|PA|A/Fox/Netherlands/EMC3/2022|EPI ISL 12069289||A / H5N1
2131816|PA|A/common tern/Germany-NI/AI03186/2022|EPI ISL 14571432||A / H5N1
1981008|PA|A/red fox/Netherlands/22002190-003/2022|EPI ISL 9616271||A / H5N1
2018064|PA|A/Fox/Netherlands/EMC1/2022|EPI ISL 12066188||A / H5N1
2297255|PA|A/Vulpes vulpes/Belgium/14775-0001/2022|EPI ISL 16549380||A / H5N1
1994442|PA|A/red fox/Netherlands/22004172-003/2022|EPI ISL 10915976||A / H5N1
2132612|PA|A/sandwich tern/Germany-MV/AI03131/2022|EPI ISL 14573037||A / H5N1
2131902|PA|A/great cormorant/Germany-MV/AI02666/2022|EPI ISL 14571448||A / H5N1
2131910|PA|A/great cormorant/Germany-MV/AI02774/2022|EPI ISL 14571449||A / H5N1
Ger-10-21-N1.5 H5N1|A/Eurasian wigeon/Germany-SH/AI05952/2021|H5N1|2021-10-14 PA
1956636|PA|A/red fox/Netherlands/22000097-004/2022|EPI ISL 8650947||A / H5N1
Ger-11-21-N1.4 H5N1|A/buzzard/Germany-SH/AI07099/2021|H5N1|2021-11-09 PA
1944252|PA|A/Eurasian wigeon/Italy/21VIR8919-3/2021|EPI ISL 7733585||A / H5N1
1945581|PA|A/Red fox/Estonia/TA2126820 21VIR10433-13/2021|EPI ISL 7778880||A / H5N1
A/Red fox/Germany/51023-60 PA
1859944|PA|A/red fox/Netherlands/21028774-002/2021|EPI ISL 2194218||A / H5N1
1921135|PA|A/barnacle goose/Germany-NI/AI03980/2021|EPI ISL 5146291||A / H5N1
1920282|PA|A/barnacle goose/Germany-NI/AI01605/2021|EPI ISL 5099456||A / H5N1
1921079|PA|A/oystercatcher/Germany-NI/AI05047/2021|EPI ISL 5145744||A / H5N1
1945089|PA|A/Eurasian curlew/Germany-SH/AI05960/2021|EPI ISL 7748001||A / H5N1
2197212|PA|A/fox/Finland/2844 21VIR9619-1/2021|EPI ISL 15518268||A / H5N1
1944230|PA|A/black-headed gull/Germany-NI/AI05962/2021|EPI ISL 7733048||A / H5N1
2197228|PA|A/otter/Finland/2860 21VIR9619-5/2021|EPI ISL 15518270||A / H5N1
2220593|PA|A/Mink/Spain/3691-8 22VIR10586-10/2022|EPI ISL 15878539||A / H5N1
2220617|PA|A/Mink/Spain/3691-3 22VIR10586-9/2022|EPI ISL 15878542||A / H5N1
2129831|PA|A/Vulpes vulpes/Belgium/9031 0008/2022|EPI ISL 14494796||A / H5N1
2540331|PA|A/Vulpes vulpes/Belgium/03117 0004/2023|EPI ISL 17606758||A / H5N1
Ger-02-23-N1.1 euBB H5N1|A/black-headed gull/Germany-BW/2023AI01419/2023|H5N1|2023-02-09 PA
2468395|PA|A/Vulpes vulpes/Belgium/01784 0010/2023|EPI ISL 17245099||A / H5N1
2468379|PA|A/Vulpes vulpes/Belgium/01784 0004/2023|EPI ISL 17244973||A / H5N1
2475127|PA|A/Black-headed gull/Netherlands/15/2023|EPI ISL 17267144||A / H5N1
2510244|PA|A/black-headed gull/Czech Republic/5152-1/2023|EPI ISL 17470105||A / H5N1
2528029|PA|A/black-headed gull/England/053469/2023|EPI ISL 17521049||A / H5N1
A/Red fox/Germany/51023-261 PA
0.01
Mammalian Europe (2021-2023)
Wild bird Europe (2021-2023)
Wild bird Germany (2021-2023)

D

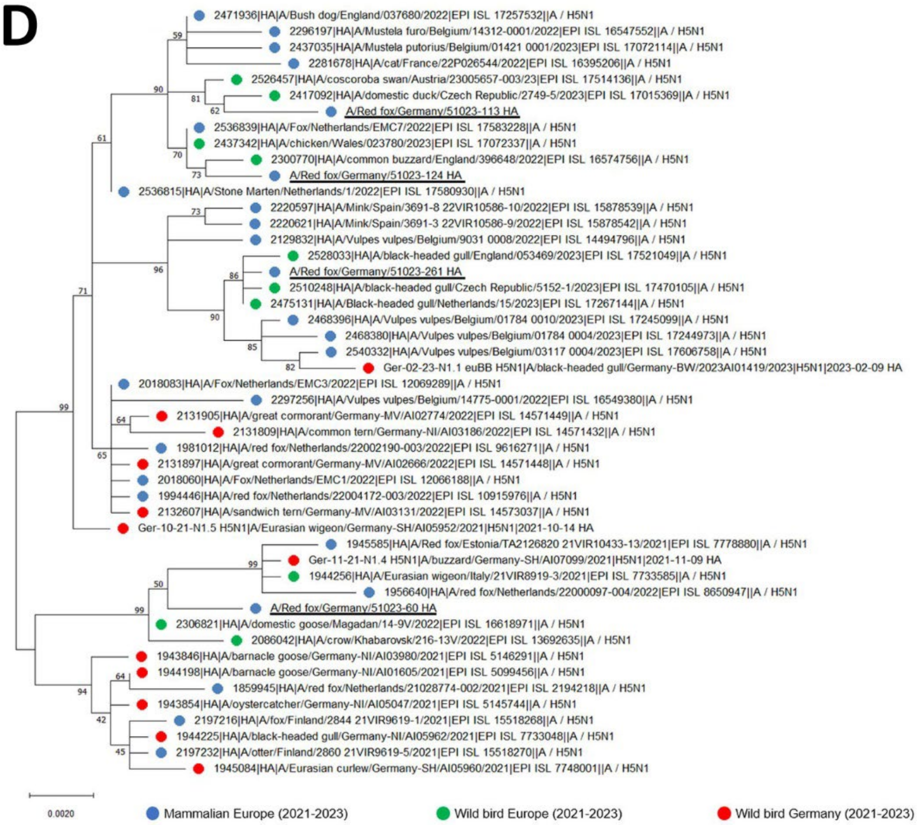
2471936|HA|A/Bush dog/England/037680/2022|EPI ISL 17257532||A / H5N1
2296197|HA|A/Mustela furo/Belgium/14312-0001/2022|EPI ISL 16547552||A / H5N1
2437035|HA|A/Mustela putorius/Belgium/01421 0001/2023|EPI ISL 17072114||A / H5N1
2281678|HA|A/cat/France/22P026544/2022|EPI ISL 16395206||A / H5N1
2526457|HA|A/coscoroba swan/Austria/23005657-003/23|EPI ISL 17514136||A / H5N1
2417092|HA|A/domestic duck/Czech Republic/2749-5/2023|EPI ISL 17015369||A / H5N1
A/Red fox/Germany/51023-113 HA
2536839|HA|A/Fox/Netherlands/EMC7/2022|EPI ISL 17583228||A / H5N1
2437342|HA|A/chicken/Wales/023780/2023|EPI ISL 17072337||A / H5N1
2300770|HA|A/common buzzard/England/396648/2022|EPI ISL 16574756||A / H5N1
A/Red fox/Germany/51023-124 HA
2536815|HA|A/Stone Marten/Netherlands/1/2022|EPI ISL 17580930||A / H5N1
2220597|HA|A/Mink/Spain/3691-8 22VIR10586-10/2022|EPI ISL 15878539||A / H5N1
2220621|HA|A/Mink/Spain/3691-3 22VIR10586-9/2022|EPI ISL 15878542||A / H5N1
2129832|HA|A/Vulpes vulpes/Belgium/9031 0008/2022|EPI ISL 14494796||A / H5N1
2528033|HA|A/black-headed gull/England/053469/2023|EPI ISL 17521049||A / H5N1
A/Red fox/Germany/51023-261 HA
2510248|HA|A/black-headed gull/Czech Republic/5152-1/2023|EPI ISL 17470105||A / H5N1
2475131|HA|A/Black-headed gull/Netherlands/15/2023|EPI ISL 17267144||A / H5N1
2468396|HA|A/Vulpes vulpes/Belgium/01784 0010/2023|EPI ISL 17245099||A / H5N1
2468380|HA|A/Vulpes vulpes/Belgium/01784 0004/2023|EPI ISL 17244973||A / H5N1
2540332|HA|A/Vulpes vulpes/Belgium/03117 0004/2023|EPI ISL 17606758||A / H5N1
Ger-02-23-N1.1 euBB H5N1|A/black-headed gull/Germany-BW/2023AI01419/2023|H5N1|2023-02-09 HA
2018083|HA|A/Fox/Netherlands/EMC3/2022|EPI ISL 12069289||A / H5N1
2297256|HA|A/Vulpes vulpes/Belgium/14775-0001/2022|EPI ISL 16549380||A / H5N1
2131905|HA|A/great cormorant/Germany-MV/AI02774/2022|EPI ISL 14571449||A / H5N1
2131809|HA|A/common tern/Germany-NI/AI03186/2022|EPI ISL 14571432||A / H5N1
1981012|HA|A/red fox/Netherlands/22002190-003/2022|EPI ISL 9616271||A / H5N1
2131897|HA|A/great cormorant/Germany-MV/AI02666/2022|EPI ISL 14571448||A / H5N1
2018060|HA|A/Fox/Netherlands/EMC1/2022|EPI ISL 12066188||A / H5N1
1994446|HA|A/red fox/Netherlands/22004172-003/2022|EPI ISL 10915976||A / H5N1
2132607|HA|A/sandwich tern/Germany-MV/AI03131/2022|EPI ISL 14573037||A / H5N1
Ger-10-21-N1.5 H5N1|A/Eurasian wigeon/Germany-SH/AI05952/2021|H5N1|2021-10-14 HA
1945585|HA|A/Red fox/Estonia/TA2126820 21VIR10433-13/2021|EPI ISL 7778880||A / H5N1
Ger-11-21-N1.4 H5N1|A/buzzard/Germany-SH/AI07099/2021|H5N1|2021-11-09 HA
1944256|HA|A/Eurasian wigeon/Italy/21VIR8919-3/2021|EPI ISL 7733585||A / H5N1
1956640|HA|A/red fox/Netherlands/22000097-004/2022|EPI ISL 8650947||A / H5N1
A/Red fox/Germany/51023-60 HA
2306821|HA|A/domestic goose/Magadan/14-9V/2022|EPI ISL 16618971||A / H5N1
2086042|HA|A/crow/Khabarovsk/216-13V/2022|EPI ISL 13692635||A / H5N1
1943846|HA|A/barnacle goose/Germany-NI/AI03980/2021|EPI ISL 5146291||A / H5N1
1944198|HA|A/barnacle goose/Germany-NI/AI01605/2021|EPI ISL 5099456||A / H5N1
1859945|HA|A/red fox/Netherlands/21028774-002/2021|EPI ISL 2194218||A / H5N1
1943854|HA|A/oystercatcher/Germany-NI/AI05047/2021|EPI ISL 5145744||A / H5N1
2197216|HA|A/fox/Finland/2844 21VIR9619-1/2021|EPI ISL 15518268||A / H5N1
1944225|HA|A/black-headed gull/Germany-NI/AI05962/2021|EPI ISL 7733048||A / H5N1
2197232|HA|A/otter/Finland/2860 21VIR9619-5/2021|EPI ISL 15518270||A / H5N1
1945084|HA|A/Eurasian curlew/Germany-SH/AI05960/2021|EPI ISL 7748001||A / H5N1
0.0020
Mammalian Europe (2021-2023)
Wild bird Europe (2021-2023)
Wild bird Germany (2021-2023)

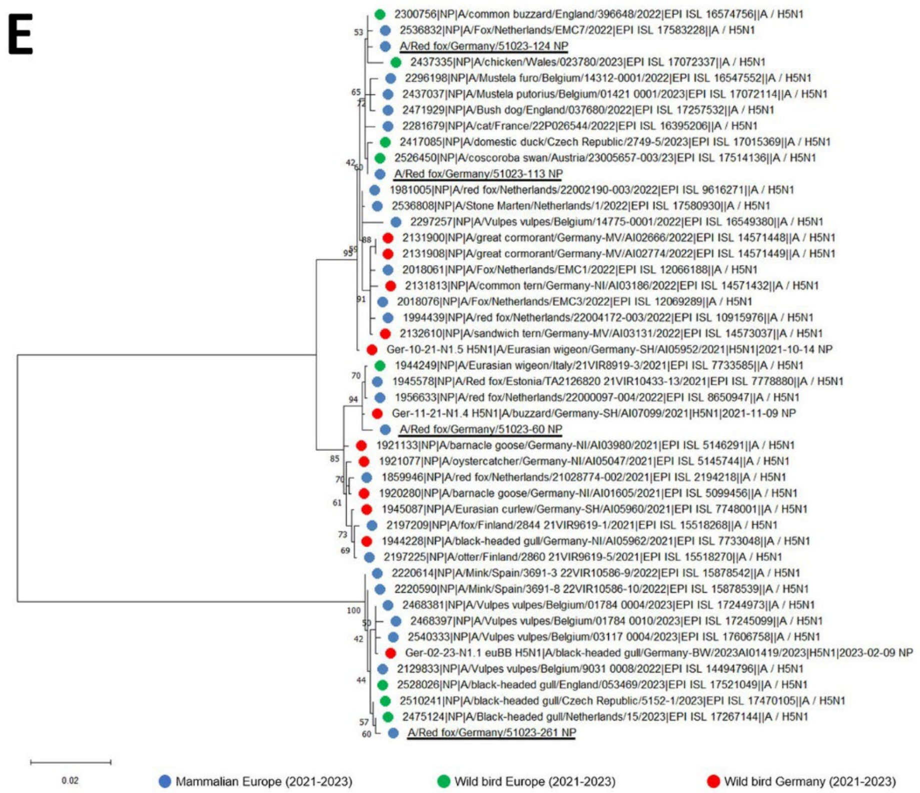
E
2300756|NP|A/common buzzard/England/396648/2022|EPI ISL 16574756||A / H5N1
2536832|NP|A/Fox/Netherlands/EMC7/2022|EPI ISL 17583228||A / H5N1
A/Red fox/Germany/51023-124 NP
2437335|NP|A/chicken/Wales/023780/2023|EPI ISL 17072337||A / H5N1
2296198|NP|A/Mustela furo/Belgium/14312-0001/2022|EPI ISL 16547552||A / H5N1
2437037|NP|A/Mustela putorius/Belgium/01421 0001/2023|EPI ISL 17072114||A / H5N1
2471929|NP|A/Bush dog/England/037680/2022|EPI ISL 17257532||A / H5N1
2281679|NP|A/cat/France/22P026544/2022|EPI ISL 16395206||A / H5N1
2417085|NP|A/domestic duck/Czech Republic/2749-5/2023|EPI ISL 17015369||A / H5N1
2526450|NP|A/coscoroba swan/Austria/23005657-003/23|EPI ISL 17514136||A / H5N1
A/Red fox/Germany/51023-113 NP
1981005|NP|A/red fox/Netherlands/22002190-003/2022|EPI ISL 9616271||A / H5N1
2536808|NP|A/Stone Marten/Netherlands/1/2022|EPI ISL 17580930||A / H5N1
2297257|NP|A/Vulpes vulpes/Belgium/14775-0001/2022|EPI ISL 16549380||A / H5N1
2131900|NP|A/great cormorant/Germany-MV/AI02666/2022|EPI ISL 14571448||A / H5N1
2131908|NP|A/great cormorant/Germany-MV/AI02774/2022|EPI ISL 14571449||A / H5N1
2018061|NP|A/Fox/Netherlands/EMC1/2022|EPI ISL 12066188||A / H5N1
2131813|NP|A/common tern/Germany-NI/AI03186/2022|EPI ISL 14571432||A / H5N1
2018076|NP|A/Fox/Netherlands/EMC3/2022|EPI ISL 12069289||A / H5N1
1994439|NP|A/red fox/Netherlands/22004172-003/2022|EPI ISL 10915976||A / H5N1
2132610|NP|A/sandwich tern/Germany-MV/AI03131/2022|EPI ISL 14573037||A / H5N1
Ger-10-21-N1.5 H5N1|A/Eurasian wigeon/Germany-SH/AI05952/2021|H5N1|2021-10-14 NP
1944249|NP|A/Eurasian wigeon/Italy/21VIR8919-3/2021|EPI ISL 7733585||A / H5N1
1945578|NP|A/Red fox/Estonia/TA2126820 21VIR10433-13/2021|EPI ISL 7778880||A / H5N1
1956633|NP|A/red fox/Netherlands/22000097-004/2022|EPI ISL 8650947||A / H5N1
Ger-11-21-N1.4 H5N1|A/buzzard/Germany-SH/AI07099/2021|H5N1|2021-11-09 NP
A/Red fox/Germany/51023-60 NP
1921133|NP|A/barnacle goose/Germany-NI/AI03980/2021|EPI ISL 5146291||A / H5N1
1921077|NP|A/oystercatcher/Germany-NI/AI05047/2021|EPI ISL 5145744||A / H5N1
1859946|NP|A/red fox/Netherlands/21028774-002/2021|EPI ISL 2194218||A / H5N1
1920280|NP|A/barnacle goose/Germany-NI/AI01605/2021|EPI ISL 5099456||A / H5N1
1945087|NP|A/Eurasian curlew/Germany-SH/AI05960/2021|EPI ISL 7748001||A / H5N1
2197209|NP|A/fox/Finland/2844 21VIR9619-1/2021|EPI ISL 15518268||A / H5N1
1944228|NP|A/black-headed gull/Germany-NI/AI05962/2021|EPI ISL 7733048||A / H5N1
2197225|NP|A/otter/Finland/2860 21VIR9619-5/2021|EPI ISL 15518270||A / H5N1
2220614|NP|A/Mink/Spain/3691-3 22VIR10586-9/2022|EPI ISL 15878542||A / H5N1
2220590|NP|A/Mink/Spain/3691-8 22VIR10586-10/2022|EPI ISL 15878539||A / H5N1
2468381|NP|A/Vulpes vulpes/Belgium/01784 0004/2023|EPI ISL 17244973||A / H5N1
2468397|NP|A/Vulpes vulpes/Belgium/01784 0010/2023|EPI ISL 17245099||A / H5N1
2540333|NP|A/Vulpes vulpes/Belgium/03117 0004/2023|EPI ISL 17606758||A / H5N1
Ger-02-23-N1.1 euBB H5N1|A/black-headed gull/Germany-BW/2023AI01419/2023|H5N1|2023-02-09 NP
2129833|NP|A/Vulpes vulpes/Belgium/9031 0008/2022|EPI ISL 14494796||A / H5N1
2528026|NP|A/black-headed gull/England/053469/2023|EPI ISL 17521049||A / H5N1
2510241|NP|A/black-headed gull/Czech Republic/5152-1/2023|EPI ISL 17470105||A / H5N1
2475124|NP|A/Black-headed gull/Netherlands/15/2023|EPI ISL 17267144||A / H5N1
A/Red fox/Germany/51023-261 NP
0.02
Mammalian Europe (2021-2023)
Wild bird Europe (2021-2023)
Wild bird Germany (2021-2023)

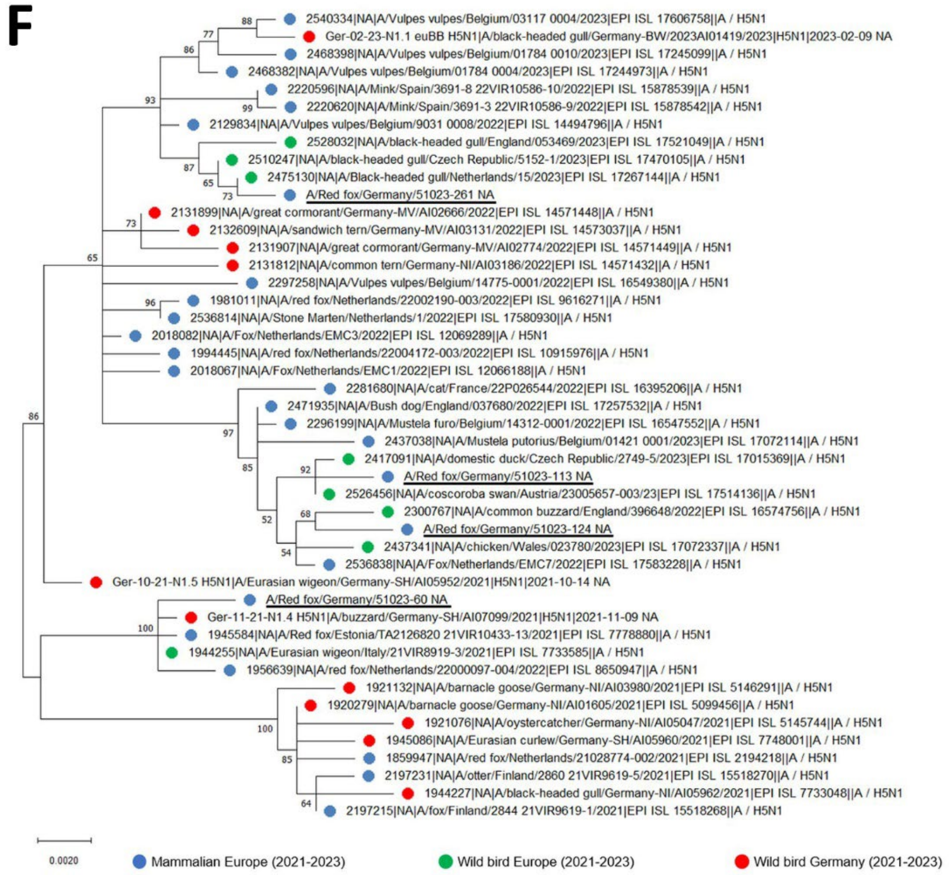
F
2540334|NA|A/Vulpes vulpes/Belgium/03117 0004/2023|EPI ISL 17606758||A / H5N1
Ger-02-23-N1.1 euBB H5N1|A/black-headed gull/Germany-BW/2023AI01419/2023|H5N1|2023-02-09 NA
2468398|NA|A/Vulpes vulpes/Belgium/01784 0010/2023|EPI ISL 17245099||A / H5N1
2468382|NA|A/Vulpes vulpes/Belgium/01784 0004/2023|EPI ISL 17244973||A / H5N1
2220596|NA|A/Mink/Spain/3691-8 22VIR10586-10/2022|EPI ISL 15878539||A / H5N1
2220620|NA|A/Mink/Spain/3691-3 22VIR10586-9/2022|EPI ISL 15878542||A / H5N1
2129834|NA|A/Vulpes vulpes/Belgium/9031 0008/2022|EPI ISL 14494796||A / H5N1
2528032|NA|A/black-headed gull/England/053469/2023|EPI ISL 17521049||A / H5N1
2510247|NA|A/black-headed gull/Czech Republic/5152-1/2023|EPI ISL 17470105||A / H5N1
2475130|NA|A/Black-headed gull/Netherlands/15/2023|EPI ISL 17267144||A / H5N1
A/Red fox/Germany/51023-261 NA
2131899|NA|A/great cormorant/Germany-MV/AI02666/2022|EPI ISL 14571448||A / H5N1
2132609|NA|A/sandwich tern/Germany-MV/AI03131/2022|EPI ISL 14573037||A / H5N1
2131907|NA|A/great cormorant/Germany-MV/AI02774/2022|EPI ISL 14571449||A / H5N1
2131812|NA|A/common tern/Germany-NI/AI03186/2022|EPI ISL 14571432||A / H5N1
2297258|NA|A/Vulpes vulpes/Belgium/14775-0001/2022|EPI ISL 16549380||A / H5N1
1981011|NA|A/red fox/Netherlands/22002190-003/2022|EPI ISL 9616271||A / H5N1
2536814|NA|A/Stone Marten/Netherlands/1/2022|EPI ISL 17580930||A / H5N1
2018082|NA|A/Fox/Netherlands/EMC3/2022|EPI ISL 12069289||A / H5N1
1994445|NA|A/red fox/Netherlands/22004172-003/2022|EPI ISL 10915976||A / H5N1
2018067|NA|A/Fox/Netherlands/EMC1/2022|EPI ISL 12066188||A / H5N1
2281680|NA|A/cat/France/22P026544/2022|EPI ISL 16395206||A / H5N1
2471935|NA|A/Bush dog/England/037680/2022|EPI ISL 17257532||A / H5N1
2296199|NA|A/Mustela furo/Belgium/14312-0001/2022|EPI ISL 16547552||A / H5N1
2437038|NA|A/Mustela putorius/Belgium/01421 0001/2023|EPI ISL 17072114||A / H5N1
2417091|NA|A/domestic duck/Czech Republic/2749-5/2023|EPI ISL 17015369||A / H5N1
A/Red fox/Germany/51023-113 NA
2526456|NA|A/coscoroba swan/Austria/23005657-003/23|EPI ISL 17514136||A / H5N1
2300767|NA|A/common buzzard/England/396648/2022|EPI ISL 16574756||A / H5N1
A/Red fox/Germany/51023-124 NA
2437341|NA|A/chicken/Wales/023780/2023|EPI ISL 17072337||A / H5N1
2536838|NA|A/Fox/Netherlands/EMC7/2022|EPI ISL 17583228||A / H5N1
Ger-10-21-N1.5 H5N1|A/Eurasian wigeon/Germany-SH/AI05952/2021|H5N1|2021-10-14 NA
A/Red fox/Germany/51023-60 NA
Ger-11-21-N1.4 H5N1|A/buzzard/Germany-SH/AI07099/2021|H5N1|2021-11-09 NA
1945584|NA|A/Red fox/Estonia/TA2126820 21VIR10433-13/2021|EPI ISL 7778880||A / H5N1
1944255|NA|A/Eurasian wigeon/Italy/21VIR8919-3/2021|EPI ISL 7733585||A / H5N1
1956639|NA|A/red fox/Netherlands/22000097-004/2022|EPI ISL 8650947||A / H5N1
1921132|NA|A/barnacle goose/Germany-NI/AI03980/2021|EPI ISL 5146291||A / H5N1
1920279|NA|A/barnacle goose/Germany-NI/AI01605/2021|EPI ISL 5099456||A / H5N1
1921076|NA|A/oystercatcher/Germany-NI/AI05047/2021|EPI ISL 5145744||A / H5N1
1945086|NA|A/Eurasian curlew/Germany-SH/AI05960/2021|EPI ISL 7748001||A / H5N1
1859947|NA|A/red fox/Netherlands/21028774-002/2021|EPI ISL 2194218||A / H5N1
2197231|NA|A/otter/Finland/2860 21VIR9619-5/2021|EPI ISL 15518270||A / H5N1
1944227|NA|A/black-headed gull/Germany-NI/AI05962/2021|EPI ISL 7733048||A / H5N1
2197215|NA|A/fox/Finland/2844 21VIR9619-1/2021|EPI ISL 15518268||A / H5N1
0.0020
Mammalian Europe (2021-2023)
Wild bird Europe (2021-2023)
Wild bird Germany (2021-2023)

G

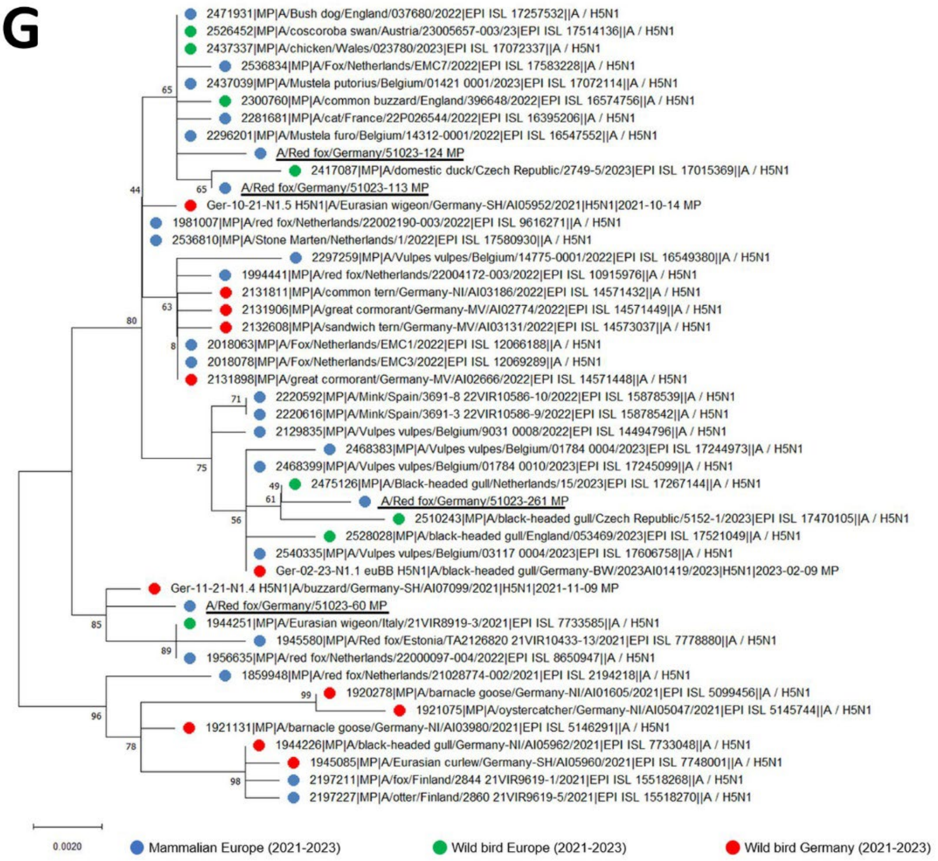
2471931|MP|A/Bush dog/England/037680/2022|EPI ISL 17257532||A / H5N1
2526452|MP|A/coscoroba swan/Austria/23005657-003/23|EPI ISL 17514136||A / H5N1
2437337|MP|A/chicken/Wales/023780/2023|EPI ISL 17072337||A / H5N1
2536834|MP|A/Fox/Netherlands/EMC7/2022|EPI ISL 17583228||A / H5N1
2437039|MP|A/Mustela putorius/Belgium/01421 0001/2023|EPI ISL 17072114||A / H5N1
2300760|MP|A/common buzzard/England/396648/2022|EPI ISL 16574756||A / H5N1
2281681|MP|A/cat/France/22P026544/2022|EPI ISL 16395206||A / H5N1
2296201|MP|A/Mustela furo/Belgium/14312-0001/2022|EPI ISL 16547552||A / H5N1
A/Red fox/Germany/51023-124 MP
2417087|MP|A/domestic duck/Czech Republic/2749-5/2023|EPI ISL 17015369||A / H5N1
A/Red fox/Germany/51023-113 MP
Ger-10-21-N1.5 H5N1|A/Eurasian wigeon/Germany-SH/AI05952/2021|H5N1|2021-10-14 MP
1981007|MP|A/red fox/Netherlands/22002190-003/2022|EPI ISL 9616271||A / H5N1
2536810|MP|A/Stone Marten/Netherlands/1/2022|EPI ISL 17580930||A / H5N1
2297259|MP|A/Vulpes vulpes/Belgium/14775-0001/2022|EPI ISL 16549380||A / H5N1
1994441|MP|A/red fox/Netherlands/22004172-003/2022|EPI ISL 10915976||A / H5N1
2131811|MP|A/common tern/Germany-NI/AI03186/2022|EPI ISL 14571432||A / H5N1
2131906|MP|A/great cormorant/Germany-MV/AI02774/2022|EPI ISL 14571449||A / H5N1
2132608|MP|A/sandwich tern/Germany-MV/AI03131/2022|EPI ISL 14573037||A / H5N1
2018063|MP|A/Fox/Netherlands/EMC1/2022|EPI ISL 12066188||A / H5N1
2018078|MP|A/Fox/Netherlands/EMC3/2022|EPI ISL 12069289||A / H5N1
2131898|MP|A/great cormorant/Germany-MV/AI02666/2022|EPI ISL 14571448||A / H5N1
2220592|MP|A/Mink/Spain/3691-8 22VIR10586-10/2022|EPI ISL 15878539||A / H5N1
2220616|MP|A/Mink/Spain/3691-3 22VIR10586-9/2022|EPI ISL 15878542||A / H5N1
2129835|MP|A/Vulpes vulpes/Belgium/9031 0008/2022|EPI ISL 14494796||A / H5N1
2468383|MP|A/Vulpes vulpes/Belgium/01784 0004/2023|EPI ISL 17244973||A / H5N1
2468399|MP|A/Vulpes vulpes/Belgium/01784 0010/2023|EPI ISL 17245099||A / H5N1
2475126|MP|A/Black-headed gull/Netherlands/15/2023|EPI ISL 17267144||A / H5N1
A/Red fox/Germany/51023-261 MP
2510243|MP|A/black-headed gull/Czech Republic/5152-1/2023|EPI ISL 17470105||A / H5N1
2528028|MP|A/black-headed gull/England/053469/2023|EPI ISL 17521049||A / H5N1
2540335|MP|A/Vulpes vulpes/Belgium/03117 0004/2023|EPI ISL 17606758||A / H5N1
Ger-02-23-N1.1 euBB H5N1|A/black-headed gull/Germany-BW/2023AI01419/2023|H5N1|2023-02-09 MP
Ger-11-21-N1.4 H5N1|A/buzzard/Germany-SH/AI07099/2021|H5N1|2021-11-09 MP
A/Red fox/Germany/51023-60 MP
1944251|MP|A/Eurasian wigeon/Italy/21VIR8919-3/2021|EPI ISL 7733585||A / H5N1
1945580|MP|A/Red fox/Estonia/TA2126820 21VIR10433-13/2021|EPI ISL 7778880||A / H5N1
1956635|MP|A/red fox/Netherlands/22000097-004/2022|EPI ISL 8650947||A / H5N1
1859948|MP|A/red fox/Netherlands/21028774-002/2021|EPI ISL 2194218||A / H5N1
1920278|MP|A/barnacle goose/Germany-NI/AI01605/2021|EPI ISL 5099456||A / H5N1
1921075|MP|A/oystercatcher/Germany-NI/AI05047/2021|EPI ISL 5145744||A / H5N1
1921131|MP|A/barnacle goose/Germany-NI/AI03980/2021|EPI ISL 5146291||A / H5N1
1944226|MP|A/black-headed gull/Germany-NI/AI05962/2021|EPI ISL 7733048||A / H5N1
1945085|MP|A/Eurasian curlew/Germany-SH/AI05960/2021|EPI ISL 7748001||A / H5N1
2197211|MP|A/fox/Finland/2844 21VIR9619-1/2021|EPI ISL 15518268||A / H5N1
2197227|MP|A/otter/Finland/2860 21VIR9619-5/2021|EPI ISL 15518270||A / H5N1
0.0020
Mammalian Europe (2021-2023)
Wild bird Europe (2021-2023)
Wild bird Germany (2021-2023)

H

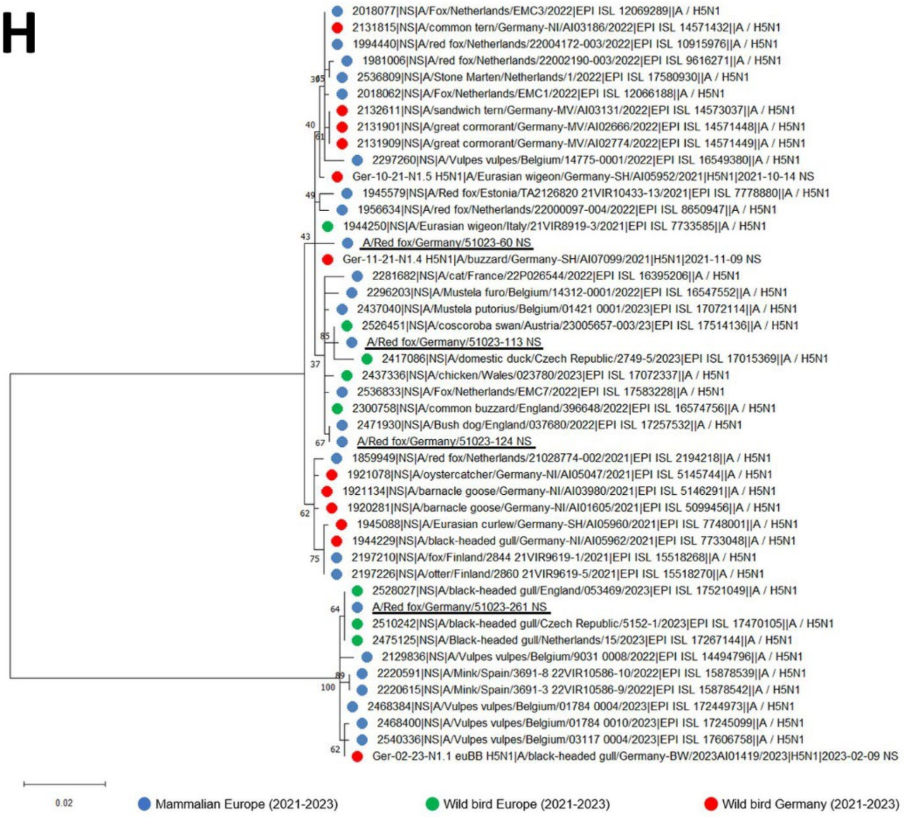
2018077|NS|A/Fox/Netherlands/EMC3/2022|EPI ISL 12069289||A / H5N1
2131815|NS|A/common tern/Germany-NI/AI03186/2022|EPI ISL 14571432||A / H5N1
1994440|NS|A/red fox/Netherlands/22004172-003/2022|EPI ISL 10915976||A / H5N1
1981006|NS|A/red fox/Netherlands/22002190-003/2022|EPI ISL 9616271||A / H5N1
2536809|NS|A/Stone Marten/Netherlands/1/2022|EPI ISL 17580930||A / H5N1
2018062|NS|A/Fox/Netherlands/EMC1/2022|EPI ISL 12066188||A / H5N1
2132611|NS|A/sandwich tern/Germany-MV/AI03131/2022|EPI ISL 14573037||A / H5N1
2131901|NS|A/great cormorant/Germany-MV/AI02666/2022|EPI ISL 14571448||A / H5N1
2131909|NS|A/great cormorant/Germany-MV/AI02774/2022|EPI ISL 14571449||A / H5N1
2297260|NS|A/Vulpes vulpes/Belgium/14775-0001/2022|EPI ISL 16549380||A / H5N1
Ger-10-21-N1.5 H5N1|A/Eurasian wigeon/Germany-SH/AI05952/2021|H5N1|2021-10-14 NS
1945579|NS|A/Red fox/Estonia/TA2126820 21VIR10433-13/2021|EPI ISL 7778880||A / H5N1
1956634|NS|A/red fox/Netherlands/22000097-004/2022|EPI ISL 8650947||A / H5N1
1944250|NS|A/Eurasian wigeon/Italy/21VIR8919-3/2021|EPI ISL 7733585||A / H5N1
A/Red fox/Germany/51023-60 NS
Ger-11-21-N1.4 H5N1|A/buzzard/Germany-SH/AI07099/2021|H5N1|2021-11-09 NS
2281682|NS|A/cat/France/22P026544/2022|EPI ISL 16395206||A / H5N1
2296203|NS|A/Mustela furo/Belgium/14312-0001/2022|EPI ISL 16547552||A / H5N1
2437040|NS|A/Mustela putorius/Belgium/01421 0001/2023|EPI ISL 17072114||A / H5N1
2526451|NS|A/coscoroba swan/Austria/23005657-003/23|EPI ISL 17514136||A / H5N1
A/Red fox/Germany/51023-113 NS
2417086|NS|A/domestic duck/Czech Republic/2749-5/2023|EPI ISL 17015369||A / H5N1
2437336|NS|A/chicken/Wales/023780/2023|EPI ISL 17072337||A / H5N1
2536833|NS|A/Fox/Netherlands/EMC7/2022|EPI ISL 17583228||A / H5N1
2300758|NS|A/common buzzard/England/396648/2022|EPI ISL 16574756||A / H5N1
2471930|NS|A/Bush dog/England/037680/2022|EPI ISL 17257532||A / H5N1
A/Red fox/Germany/51023-124 NS
1859949|NS|A/red fox/Netherlands/21028774-002/2021|EPI ISL 2194218||A / H5N1
1921078|NS|A/oystercatcher/Germany-NI/AI05047/2021|EPI ISL 5145744||A / H5N1
1921134|NS|A/barnacle goose/Germany-NI/AI03980/2021|EPI ISL 5146291||A / H5N1
1920281|NS|A/barnacle goose/Germany-NI/AI01605/2021|EPI ISL 5099456||A / H5N1
1945088|NS|A/Eurasian curlew/Germany-SH/AI05960/2021|EPI ISL 7748001||A / H5N1
1944229|NS|A/black-headed gull/Germany-NI/AI05962/2021|EPI ISL 7733048||A / H5N1
2197210|NS|A/fox/Finland/2844 21VIR9619-1/2021|EPI ISL 15518268||A / H5N1
2197226|NS|A/otter/Finland/2860 21VIR9619-5/2021|EPI ISL 15518270||A / H5N1
2528027|NS|A/black-headed gull/England/053469/2023|EPI ISL 17521049||A / H5N1
A/Red fox/Germany/51023-261 NS
2510242|NS|A/black-headed gull/Czech Republic/5152-1/2023|EPI ISL 17470105||A / H5N1
2475125|NS|A/Black-headed gull/Netherlands/15/2023|EPI ISL 17267144||A / H5N1
2129836|NS|A/Vulpes vulpes/Belgium/9031 0008/2022|EPI ISL 14494796||A / H5N1
2220591|NS|A/Mink/Spain/3691-8 22VIR10586-10/2022|EPI ISL 15878539||A / H5N1
2220615|NS|A/Mink/Spain/3691-3 22VIR10586-9/2022|EPI ISL 15878542||A / H5N1
2468384|NS|A/Vulpes vulpes/Belgium/01784 0004/2023|EPI ISL 17244973||A / H5N1
2468400|NS|A/Vulpes vulpes/Belgium/01784 0010/2023|EPI ISL 17245099||A / H5N1
2540336|NS|A/Vulpes vulpes/Belgium/03117 0004/2023|EPI ISL 17606758||A / H5N1
Ger-02-23-N1.1 euBB H5N1|A/black-headed gull/Germany-BW/2023AI01419/2023|H5N1|2023-02-09 NS
0.02
Mammalian Europe (2021-2023)
Wild bird Europe (2021-2023)
Wild bird Germany (2021-2023)

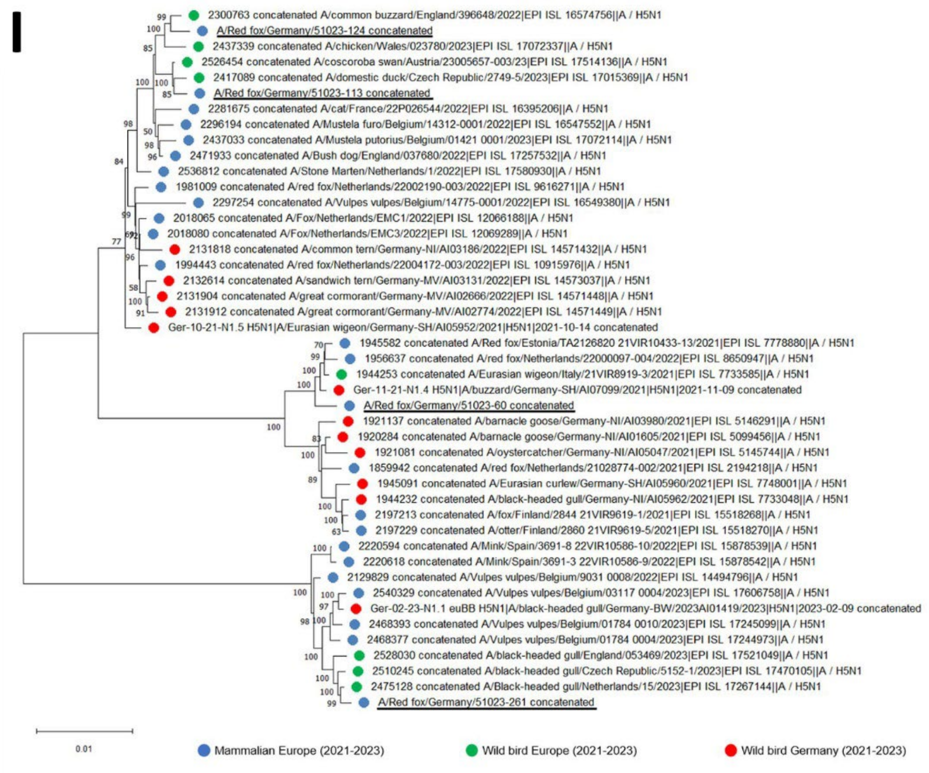

Appendix Figure. Phylogenetic maximum-likelihood analyses of all 8 HPAIV H5 segmental sequences and concatenated genomes of mammals and wild birds with 500 bootstrap iterations. Virus variants found in this study are underlined. Numbers along branches indicate percentage bootstrap values. Scale bars indicate nucleotide substitutions per site. A) polymerase basic 2; B) polymerase basic 1; C) polymerase acidic; D) hemagglutinin; E) nucleoprotein; F) neuraminidase; G) matrix; H, nonstructural protein; I) concatenated.
